# Finding of Agr Phase Variants in Staphylococcus aureus

**DOI:** 10.1128/mBio.00796-19

**Published:** 2019-08-06

**Authors:** Vishal Gor, Aya J. Takemura, Masami Nishitani, Masato Higashide, Veronica Medrano Romero, Ryosuke L. Ohniwa, Kazuya Morikawa

**Affiliations:** a Graduate School of Comprehensive Human Sciences, University of Tsukuba, Tsukuba, Japan; b Human Biology Program, School of Integrative and Global Majors, University of Tsukuba, Tsukuba, Japan; c Kotobiken Medical Laboratories, Inc., Tsukuba, Japan; d Division of Biomedical Science, Faculty of Medicine, University of Tsukuba, Tsukuba, Japan; e Center for Biotechnology, National Taiwan University, Taipei, Taiwan; University of Colorado School of Medicine and the Denver VA Healthcare System; Nanyang Technological University

**Keywords:** Agr, phase variant, *Staphylococcus aureus*

## Abstract

Staphylococcus aureus is an important human pathogen whose success is largely attributed to its vast arsenal of virulence factors that facilitate its invasion into, and survival within, the human host. The expression of these virulence factors is controlled by the quorum sensing accessory gene regulator (Agr) system. However, a large proportion of clinical S. aureus isolates are consistently found to have a mutationally inactivated Agr system. These mutants have a survival advantage in the host but are considered irreversible mutants. Here we show, for the first time, that a fraction of Agr-negative mutants can revert their Agr activity. By serially passaging Agr-negative strains and screening for phenotypic reversion of hemolysis and subsequent sequencing, we identified two mutational events responsible for reversion: a genetic duplication plus inversion event and a poly(A) tract alteration. Additionally, we demonstrate that one clinical Agr-negative methicillin-resistant S. aureus (MRSA) isolate could reproducibly generate Agr-revertant colonies with a poly(A) tract genetic mechanism. We also show that these revertants activate their Agr system upon phagocytosis. We propose a model in which a minor fraction of Agr-negative S. aureus strains are phase variants that can revert their Agr activity and may act as a cryptic insurance strategy against host-mediated stress.

## Abstract (precis)

Staphylococcus aureus is responsible for a broad range of infections. This pathogen has a vast arsenal of virulence factors at its disposal, but avirulent strains are frequently isolated as the cause of clinical infections. These isolates have a mutated *agr* locus and have been believed to have no evolutionary future. Here we show that a fraction of Agr-negative strains can repair their mutated *agr* locus with mechanisms resembling phase variation. The *agr* revertants sustain an Agr OFF state as long as they exist as a minority but can activate their Agr system upon phagocytosis. These revertant cells might function as a cryptic insurance strategy to survive immune-mediated host stress that arises during infection.

## INTRODUCTION

Staphylococcus aureus is a Gram-positive coccoid bacterium that inhabits human skin surfaces and nasal cavities as a commensal organism (1, 2). However, it is also an opportunistic pathogen and can cause diverse infections ranging from superficial skin abscesses to severe bacteremia and toxic shock syndrome (3). With its rapid development of antimicrobial resistance, S. aureus is a major health burden on a global scale (4).

One of the key contributors to the effectiveness of S. aureus as a pathogen is its vast arsenal of virulence factors that facilitate the establishment of infection (5). The transition from a commensal to a pathogenic state is driven by the expression of these virulence factors, and thus, they are tightly controlled by the accessory gene regulator (Agr) system. The *agr* locus consists of the RNAII and RNAIII transcriptional units that are under the control of the P2 and P3 promoters, respectively (see Fig. S1 in the supplemental material). The RNAII operon consists of genes responsible for a quorum-dependent autoinducing circuit (*agrBDCA*) that positively drives its own expression as well as that of the RNAIII unit. RNAIII encodes δ hemolysin and also functions as a regulatory RNA that promotes, both directly and indirectly, the expression of several exotoxins and suppresses that of colonizing factors, thus enabling S. aureus to switch to a pathogenic lifestyle (6). The switch of Agr from an “OFF” to “ON” state is not immediate, and it has been shown that a heterogeneous population will transiently exist during the gradual activation of the system, which may play a role in the infection process (7).

10.1128/mBio.00796-19.1 FIG S1 Basic architecture of the Agr quorum sensing system. The *agr* locus is composed of two divergently transcribed units: the *agrBDCA* operon under the control of the P2 promoter and the *RNAIII/hld* gene under the control of the P3 promoter. The AgrD precursor peptide undergoes two modifications to convert it into mature autoinducing peptide (AIP). The first modification is mediated by the transmembrane AgrB and involves cleavage of the C-terminal recognition sequence and formation of the thiolactone ring. This step is energetically unfavorable, and the reaction is driven by rapid degradation of the cleaved C-terminal fragment. The thiolactone intermediate is then exported out of the cell and further cleaved at the N terminus to give the mature AIP molecule. When the extracellular concentration of AIP reaches a threshold, it is detected by the AgrC receptor histidine kinase (RHK), which results in self-phosphorylation of AgrC and subsequent transfer of the phosphate group to the AgrA response regulator (RR). Phosphorylated AgrA then binds, with differential affinity, to the P2 and P3 promoters to initiate transcription. Transcription from the P3 promoter produces the RNAIII regulatory mRNA, which has global effects on gene expression and also encodes the δ hemolysin gene, while transcription from the P2 promoter creates a positive-feedback loop. Download FIG S1, TIF file, 13.0 MB. Copyright © 2019 Gor et al. 2019 Gor et al. This content is distributed under the terms of the Creative Commons Attribution 4.0 International license.

Interestingly, although the Agr system plays critical roles in virulence regulation, it is known to be genetically unstable and there are frequent reports of Agr-negative mutants being isolated from both the clinical and laboratory settings (8–12). In particular, Traber et al. observed that approximately 22% (33 out of 146) of S. aureus-positive primary cultures were completely composed of Agr-negative strains (8). There is some evidence to suggest that Agr-negative strains exhibit greater fitness, especially under antibiotic stress (13). Studies have also shown that Agr-negative strains are linked to higher patient mortality as well as increased duration of bacteremia (11, 14) and are thus considered to be making an evolutionary trade-off (15). Mutations responsible for Agr-negative phenotypes usually are located in the *agrA* and *agrC* hot spots with known Agr shutdown mutations, including frameshift insertion/deletions, nonsynonymous single nucleotide polymorphisms (SNPs), and poly(A) tract alterations (10, 12, 16, 17). The *agr* mutants isolated from patients usually have single mutations, suggesting that they are short-lived (12, 15), and the current understanding is that these mutants are evolutionarily irreversible derivatives which become extinct.

In bacteria, phase variation is known to be an evolutionarily sustainable mechanism of high-frequency gene expression switching (usually >10^−5^ per generation) and plays a role in adaptation to different environments (18). The first known report describing prokaryotic phase variation was published as far back as 1922 for *Salmonella* spp. (19), and later work identified the responsible mechanism as a site-specific genomic rearrangement (20). Another mechanism of phase variation is slipped-strand mispairing (SSM) in short sequence repeat (SSR) regions. Depending on their location, SSMs in SSRs can lead to changes in transcription or in translation (21). A documented example of SSM-mediated phase variation in S. aureus occurs in the immune-evasion-related *mapW* gene, where alterations in a poly(A) tract within the open reading frame lead to variability in MapW expression (22). Some mechanisms offer more control in switching. An example is a switch to a biofilm-negative phenotype in S. aureus resulting from disruption of biofilm-associated genes by insertion sequence 256 (IS*256*), and the transposition is negatively regulated by the sigma factor σ^B^ (23, 24).

Agr dysfunction in the strain RN4200 is due to a change in the number of successive adenines at the 3′ end of the *agrA* coding region, reminiscent of poly(A) tract phase variation (16), but to date the reversibility had not been confirmed. In this study, we aimed to prove that a fraction of Agr-negative mutants are phase variants and would thus retain the ability to revert their Agr activity. To this end, we first generated *in vitro* Agr-negative variants using the S. aureus strains MW2 and s0437. We subjected the variants to successive subcultures before screening for reversion of Agr activity. We were able to isolate Agr-positive colonies from the Agr-negative variants of both strains in this manner. Furthermore, sequencing of the Agr locus of the phenotypically reversible strains identified a genetic mechanism underlying the observed reversibility. In addition, we identified one clinical methicillin-resistant S. aureus (MRSA) isolate, designated RAV(66r), that gave rise to Agr revertant colonies. To our knowledge, this is the first report describing that a portion of Agr-negative variants can revert their Agr activity. This study adds a new perspective to the lifestyle of S. aureus, in particular the aspect concerning Agr shutdown tactics, and broadens our understanding of its adaptability.

## RESULTS

### Serial passaging of S. aureus generates Agr-negative variants.

By their nature, phase variants emerge spontaneously over time with successive generations of growth. To isolate *in vitro* Agr-negative S. aureus, we serially passaged strains MW2 and s0437 and screened for nonhemolytic colonies on sheep blood agar (SBA). Hemolysis on SBA was used as the first screen, as it provided a quick method to distinguish potential Agr-negative colonies from a large number of samples. MW2 and s0437 were passaged in tryptic soy broth (TSB), TSB containing 5% human blood, or TSB containing 5% human serum. Nonhemolytic colonies did not appear in some experiments even after several passages, but in others a large number were detected. Namely, the emergence of nonhemolytic colonies fluctuated greatly due to their spontaneous nature. All growth conditions allowed for generation of nonhemolytic variants. In total, 209 out of 14,948 MW2 colonies and 4 out of the 308 s0437 colonies were nonhemolytic.

### Agr-negative variants can phenotypically and genotypically revert to the wild type.

**FIG 1:**
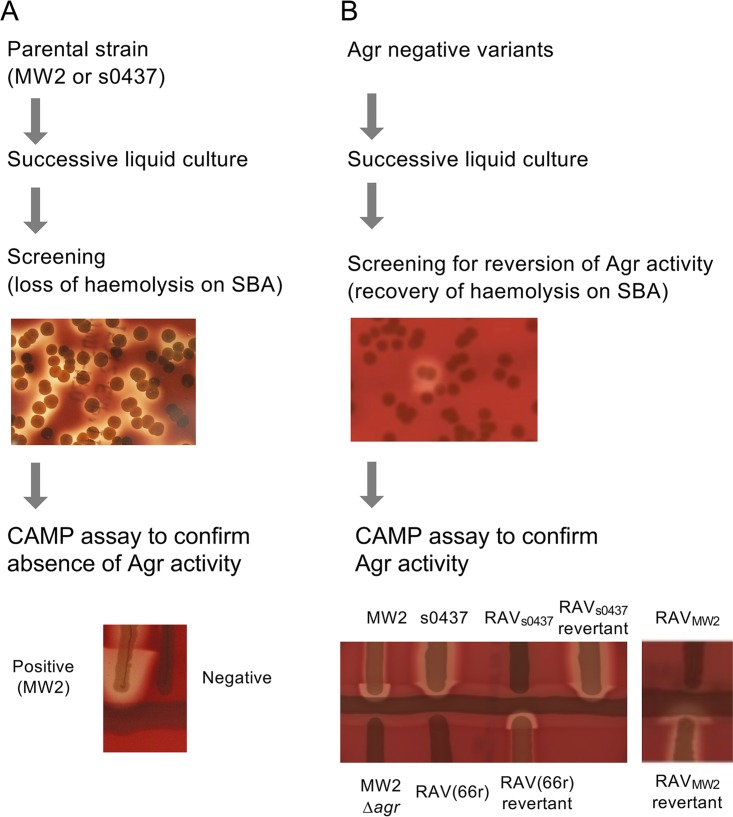
Serial passaging of Agr-negative strains can give rise to colonies with reverted Agr activity. Shown are flowcharts describing the screening procedure used to isolate strains in this study. (A) Parental strains MW2 and s0437 were passaged in successive liquid cultures, followed by screening for nonhemolytic colonies. CAMP tests were carried out on the nonhemolytic colonies alongside the parent strains to confirm loss of Agr activity. The CAMP image depicts a nonhemolytic isolate generated from MW2. (B) Agr-negative variants were passaged in successive liquid cultures, followed by screening for hemolytic colonies. CAMP tests were carried out on hemolytic colonies alongside the reversible Agr-variant (RAV) strains and the original parental strains. The CAMP images depict RAV_MW2_ and RAV_s0437_ alongside their respective revertant and WT stains. The Agr-negative MRSA clinical isolate RAV(66r) and the RAV(66r) revertant are also shown. MW2 Δ*agr* was used as a negative control.

We then tested if the isolated Agr-negative variants could revert their Agr activity over multiple generations. We carried out successive passages of 30 isolated Agr-negative variants and plated serial dilutions of the cultures onto SBA to check for the generation of any hemolytic colonies. Two *agr*-negative variants {termed RAV_MW2_ (reversible Agr variant [AV] derived from MW2) and RAV_s0437_ (from s0437)} were able to reproducibly give rise to hemolytic colonies. To verify that these colonies were Agr revertants, we carried out CAMP tests and confirmed the presence of the characteristic δ hemolysin arrowhead in the revertant colonies that was absent in the Agr-negative variants (Fig. 1). The average frequencies of revertants after 4 successive cultures (i.e., the number of hemolytic colonies compared to nonhemolytic colonies on SBA plates) were ∼2% in RAV_MW2_ and ∼0.8% in RAV_s0437_ (Table 1). We also noted that RAV_MW2_ generated 4-fold-higher numbers of rifampin-resistant colonies (median = 2.3 × 10^−8^ and mean = 2.7 × 10^−8^ [*n* = 10]) after one overnight culture than did MW2 (median = 5.4 × 10^−9^ and mean = 6.8 × 10^−9^ [*n* = 8]) (*P* < 0.01, in a two-tailed *t* test), although this study did not further address if the higher mutation rate is responsible for the reversion rate of RAV_MW2_. The background mutation rates (measured by rifampin resistance) in the s0437 wild-type (WT) strain (median = 1.4 × 10^−7^ and mean = 1.4 × 10^−7^ [*n* = 10]) and RAV_s0437_ (median = 1.0 × 10^−7^ and mean = 9.7 × 10^−8^ [*n* = 10]) were also determined.

**TABLE 1:** Summary of Agr variant and revertant strains generated and collected in this study

Strain name	Phenotype	Original strain	Culture condition	Revertant frequency, mean ± SD (%) (no. of expts)
AV1_MW2_	Nonhemolytic	MW2	TSB	
AV2_MW2_	Nonhemolytic	MW2	TSB	
AV3_MW2_	Nonhemolytic	MW2	TSB	
AV4_MW2_	Nonhemolytic	MW2	TSB	
RAV_MW2_	Nonhemolytic	MW2	TSB	2.07 ± 0.68 (10)
RAV_MW2_ revertant	Hemolytic	RAV_MW2_	TSB	
RAV_s0473_	Nonhemolytic	s0473	TSB + 5% blood	0.82 ± 0.22 (10)
RAV_s0473_ revertant	Hemolytic	RAV_s0473_	TSB	
RAV(66r)	Nonhemolytic			0.12 ± 0.08 (6)
RAV(66r) revertant	Hemolytic	RAV(66r)	TSB	

**FIG 2:**
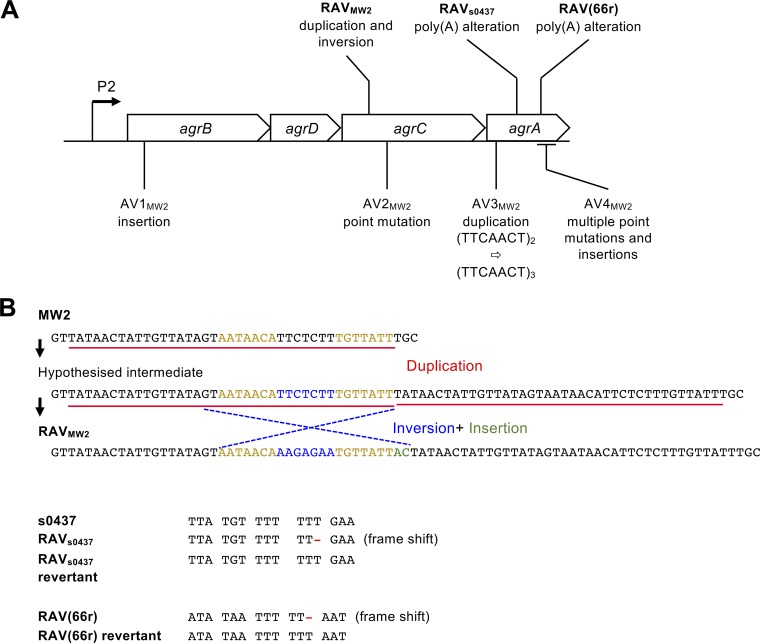
Sequence diagram of MW2 variants and AT2 revertant. (A) A map of the *agr* P2 locus showing the location of the changes identified in the reversible and irreversible Agr mutants generated or collected in this study. The duplication event identified in AV3_MW2_ is also depicted. (B) A cartoon depicting the inversion and duplication event inferred for RAV_MW2_ as well as the affected sequences in RAV_s0437_ and the RAV(66r) revertant.

Sequencing of the *agr* loci of the variants (both the reversible isolates and some of the irreversible isolates) and revertants uncovered underlying genetic mechanisms for Agr shutdown (Fig. 2). RAV_MW2_ had a duplication and inversion in *agrC*. The *agr* locus in the RAV_MW2_ revertant was identical to that of the WT. RAV_s0437_ had an alteration in a poly(A) tract within *agrA*, which reverted to the WT sequence in the RAV_s0437_ revertant. Most of the sequenced irreversible Agr-negative MW2 isolates had insertions and/or point mutations across the *agr* locus, in line with previous studies (8–10). Although one irreversible isolate (AV3_MW2_) had a duplication within *agrA*, we were unable to generate any revertants. Taken together, these results show that a fraction of the Agr-negative strains isolated *in vitro* can phenotypically revert their Agr activity and that there are multiple underlying mutational events behind this reversion.

We also carried out the revertant generation procedure on 61 clinically isolated Agr-negative strains. Although some strains showed a phenotypic Agr reversion in some experiments, only one strain, an MRSA isolate designated RAV(66r), was reproducibly reversible (Table 1). However, the detection limit of the initial screening was ∼3.0 × 10^−3^, and thus, the possibility remains that more reproducibly reversible strains could be identified using a higher detection limit. The mean frequency of reversion in RAV(66r) was 0.12% (Table 1). Upon sequencing of the *agrA* and -*C* loci of RAV(66r) and the RAV(66r) revertant, a reversible poly(A) tract alteration was found in *agrA* (Fig. 2). The average background mutation rate for RAV(66r) was 7.0 × 10^−9^ (median = 5.4 × 10^−9^ [*n* = 10]).

A summary of all the Agr variants and revertants generated or collected in this study, along with the growth conditions used for their generation as well as the origin strain, can be found in Table 1.

### A minor population of *agr*-intact cells does not activate its own Agr system in a non-autoinducing peptide (AIP)-producing population but can through local segregation on solid media.

Due to its quorum sensing nature and the low reversion frequencies, it is expected that the minor population of revertant cells emerging from the Agr-negative population in a liquid culture would sustain the Agr OFF state. Revertant cells may be able to activate their Agr system on solid structured medium, as it allows for segregation of the revertant cells into a discrete locality.

**FIG 3:**
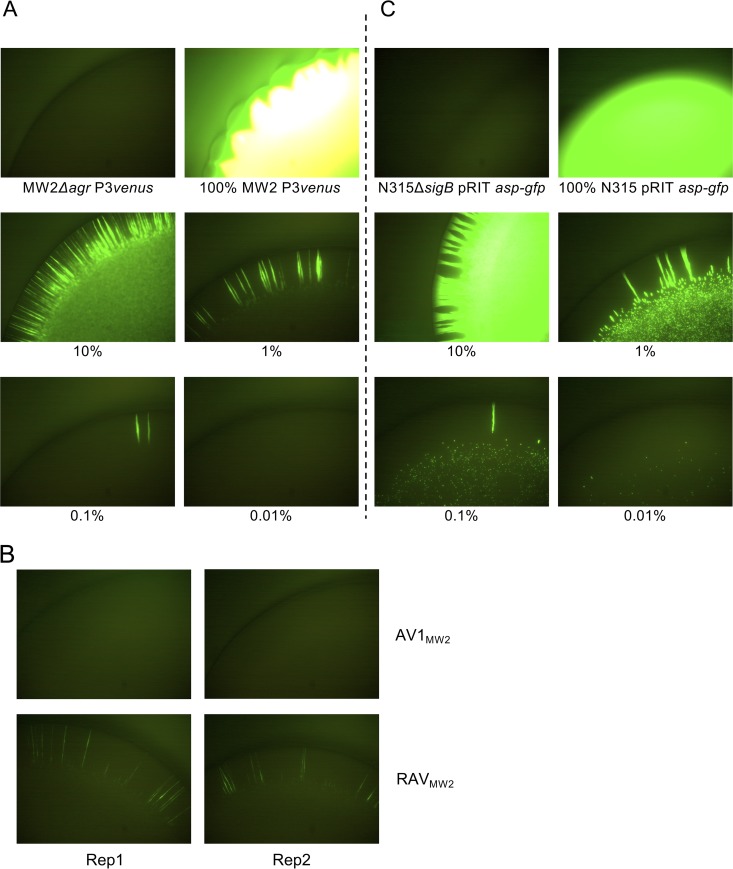
Agr intact cells do not activate their Agr system as a minority unless they undergo local segregation. Shown are representative fluorescence images of colonies of MW2 P3*venus* and mixtures of 10%, 1%, 0.1%, and 0.01% MW2 P3*venus* in MW2 Δ*agr* P3*venus* (A) or N315 pRIT*asp-gfp* and mixtures of 10%, 1%, 0.1%, and 0.01% N315 pRIT*asp-gfp* in N315 *ΔsigB* pRIT*asp-gfp* (C). Mixtures were spotted onto TSA and grown overnight before fluorescence was viewed under a stereomicroscope. (B) Representative fluorescence images of colonies of the irreversible AV1_MW2_ and reversible RAV_MW2_ Agr variants on TSA. Two independent observations are shown (Rep1 and Rep2).

To test this, Agr activity was monitored using a *venus* reporter system under the control of the *agr* P3 promoter in populations of an isogenic Δ*agr* strain unable to produce any AIP (MW2 *Δagr* P3*venus*) with various percentages of *agr*-intact cells (MW2 P3*venus*) mixed in, as well as in populations of the reversible RAV_MW2_. Venus fluorescent protein is stable enough to detect even transient activation of a promoter at the endpoint of the experiment. When spotted onto TSA with 12.5 μg/ml of chloramphenicol (Cm12.5), no fluorescence was observed in the spotted areas of samples with an MW2 P3*venus* content less than 10% (Fig. 3A). However, we were able to visualize streaks of localized fluorescence on the outer edges of spotted areas in samples with an MW2 P3*venus* content as low as 0.1% (Fig. 3A). This suggests that a minor population of *agr*-intact cells can activate their Agr system upon propagation on solid media, likely because the AIP concentration in the locality can exceed the threshold. A similar pattern of fluorescence could be detected in RAV_MW2_ but not in the irreversible strain AV1_MW2_ (Fig. 3B). RAV_MW2_, which carries a mutation in *agrC*, would still be able to produce basal levels of AIP, but the local signals in the RAV_MW2_ colony suggested the presence of a minor population of *agr*-intact revertant that were generated through colony expansion.

Figure 3C shows a control reporter system expressing *gfp* under the control of the SigB-dependent *asp* promoter in N315 and its isogenic Δ*sigB* strain. Green fluorescent protein (GFP)-positive cells remained detectable within the entire spotted area of samples with low N315 content (Fig. 3C), confirming that the quorum-independent reporter system can be detected in the same experimental settings.

**FIG 4:**
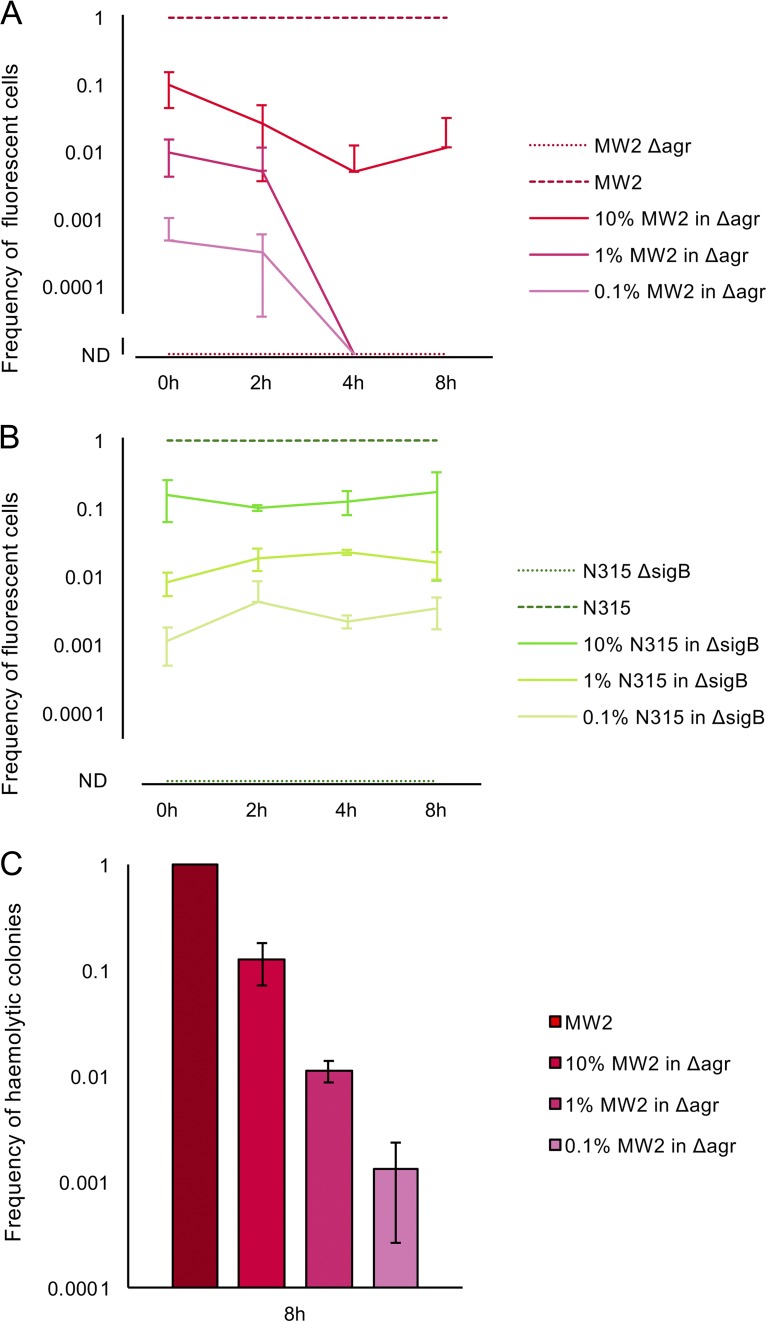
Agr-intact cells do not activate their Agr system when they are a minority in an Agr-negative population growing in liquid culture. (A) Frequency of fluorescent cells in liquid cultures of MW2 P3*venus*, MW2 Δ*agr* P3*venus*, or mixtures of 10%, 1%, and 0.1% MW2 P3*venus* in MW2 Δ*agr* P3*venus*. Aliquots were taken at 0, 2, 4, and 8 h for microscopic analysis. (B) Frequency of fluorescent cells in liquid cultures of N315 pRIT*asp-gfp*, N315 *ΔsigB* pRIT*asp-gfp*, or mixtures of 10%, 1%, and 0.1% N315 pRIT*asp-gfp* in N315 *ΔsigB* pRIT*asp-gfp*. Aliquots were taken at 0, 2, 4, and 8 h for microscopic analysis. (C) Frequency of hemolytic colonies on SBA. Samples from panel A were taken at the 8-h time point and spread onto SBA to confirm the continued presence of Agr-positive cells.

When the proportion of Agr-positive cells in liquid culture corresponded to the observed reversion frequency, i.e., <10%, reporter activity was undetectable during the time course tested (Fig. 4A). In contrast, when the proportion of WT cells was increased to 10%, reporter expression was observed, suggesting that this is the threshold that is necessary for Agr activation under planktonic conditions. The frequency of SigB active cells remained constant and detectable for all samples over the time course tested (Fig. 4B). To eliminate the possibility that the *agr*-intact cells had been lost during the time course tested, we plated the samples on SBA after the experiment and confirmed that the frequency of hemolytic cells had not changed from the start of the experiment (Fig. 4C).

Taken together, these data indicate that a minor population of Agr revertant cells does not activate its Agr system when growing in planktonic conditions.

### Phagocytosis triggers expression of the Agr system in revertant cells.

We showed that *agr*-intact cells sustain the Agr OFF state when they are a minority (<10%) in an Agr-negative population. It is known that a single *agr*-intact cell can activate its own Agr system when it is packed into a narrow matrix mimicking the phagosome environment (25) in a phenomenon termed “diffusion sensing” (26, 27) and that Agr activation is induced upon phagocytosis (28, 29). Therefore, the phagosome environment must allow *agr* revertant cells to switch the Agr system ON.

**FIG 5:**
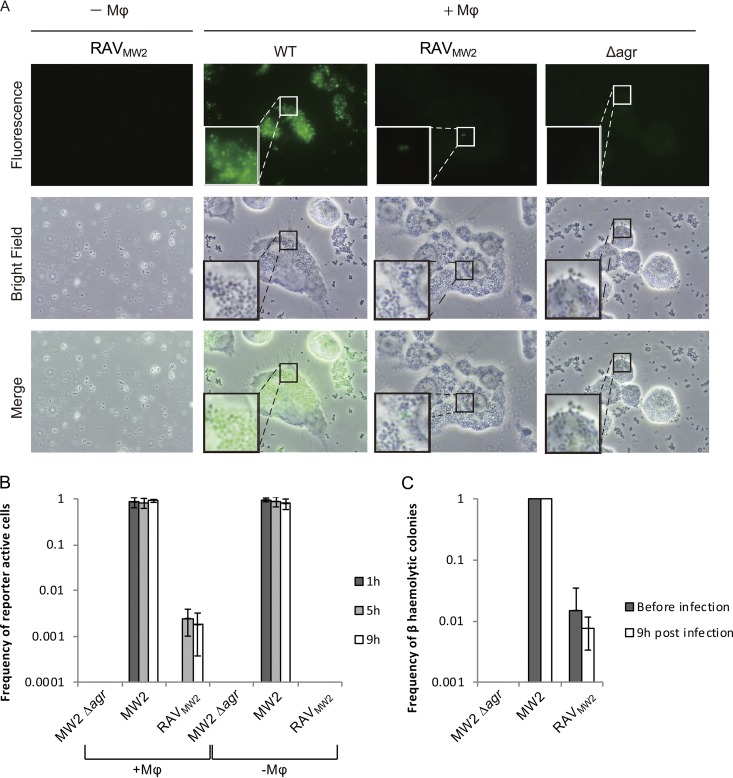
Agr-revertant cells can activate their Agr system in macrophages. (A) Representative fluorescence microscopy images of MW2 P3*venus*, RAV_MW2_ P3*venus*, and the MW2 Δagr P3*venus* in the presence and absence of macrophages (Mφ) at 9 h postinfection. (B) Average frequencies of reporter active cells from 3 independent Mφ experiments. (C) Frequency of hemolytic colonies on SBA from samples spread before and after exposure to macrophages.

To test this, a phagocytosis assay on RAV_MW2_ P3*venus* was carried out. An RAV_MW2_ overnight culture contained minor revertant cells (Fig. 5C, RAV_MW2_, Before infection). These minor revertant cells were negative in GFP expression in liquid culture without macrophages (Fig. 5A, −Mϕ). As expected, fluorescent cells were detected within macrophages at 4 and 8 h postinfection (Fig. 5A and B). To test whether the presence of macrophages affected the *agr* reversion frequency, serial dilutions of RAV_MW2_P3*venus* were plated on SBA before and after infection. The frequencies of *agr* revertant hemolytic colonies were not significantly different between the two time points, indicating that macrophages do not affect *agr* reversion (Fig. 5C). Taken together, these results suggest that revertant cells activate their *agr* system when in the confined environment of the phagosome and suggest a possible function of revertant cells.

## DISCUSSION

**FIG 6:**
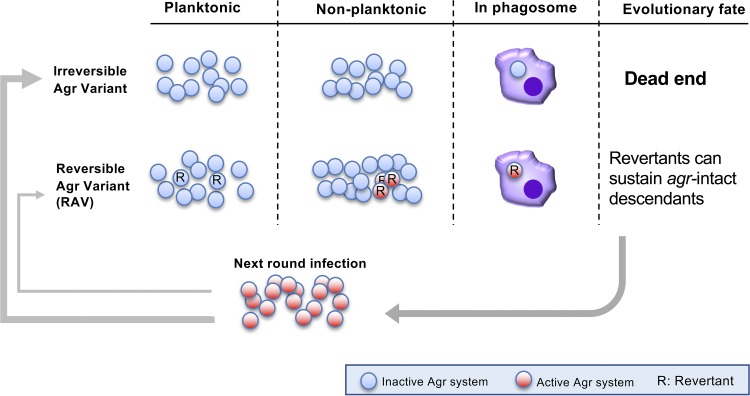
Summary of the behavior of reversible Agr variants (RAVs) and hypothetical relevance of RAVs in the staphylococcal life cycle. Under planktonic conditions, RAVs and the revertants (designated R) are phenotypically indistinguishable from irreversible variants and share the common benefits of an Agr OFF characteristic as a population. However, revertant cells can regain their Agr activity through nonplanktonic growth or upon challenge by macrophages. While the irreversible variant appears to be the majority in the staphylococcal life (infection) cycle, the minor RAVs might constitute an important strategy to maintain *agr* intact species while elegantly concealing their Agr activity during chronic infection. Mixed populations of dead-end and RAVs may also exist in some infections, but this has not yet been elucidated.

In this study, we showed that a part of Agr-negative isolates generated *in vitro*, as well as those isolated from patients, can phenotypically revert their Agr activity with underlying genetic mechanisms reminiscent of phase variation. In addition, we showed that while revertant cells are generated at a low frequency, allowing them to sustain an Agr OFF state in planktonic culture, they can activate their Agr system upon phagocytosis. This suggests a cryptic strategy for the bacterial population to survive phagocytic stress and allow for continued infection (Fig. 6).

Agr-negative strains are commonly isolated from patients and are linked to more severe infection outcomes, being associated with higher mortality and increased duration of bacteremia (11, 14). In fact, Agr-negative strains have been shown to have a higher propensity in causing bacteremia, and this has been suggested as being due to a lower energy burden (13) conferring a fitness increase (15). In general, infections arising from Agr-negative S. aureus are considered a “dead end,” as interhost spread of the bacteria is impaired and thus the infecting strain lineage goes extinct (12, 15). According to the “evolutionary trade-offs” concept proposed by Laabei et al., highly toxic S. aureus isolates that tend to be associated with superficial skin and soft tissue infections have high interhost fitness and are maintained at the epidemiological level, while low-toxicity isolates that have propensity to cause invasive infections (bacteremia) are impaired in interhost spread (15). On the other hand, recent studies carried out in mouse models suggest that Agr-negative S. aureus can spread between hosts via the gastrointestinal tract (30), and thus, Agr dysfunction may result in complexity, rather than outright abolishment, of passage to the next host. It is interesting that non-toxin-producing strains have also been linked to the shift from commensalism to infection (15, 31). In this context, the phase variation-mediated shutdown of the Agr system adds an extra layer of complexity for S. aureus.

Neutrophils and macrophages are key elements in the host innate immune response to S. aureus infection. It has long been known that S. aureus can survive within phagosomes of these immune cells in an Agr-dependent manner (28, 29) without compromising the viability of the phagocytes through upregulation of antiapoptotic factors (32, 33). There are suggestions that phagocytic cells may act as a “Trojan horse” by facilitating dissemination of S. aureus to different infectious sites (34–36). Gresham et al. showed that S. aureus-containing polymorphonuclear neutrophils can successfully establish infection in naive mice (37), and Lehar et al. demonstrated that intracellular MRSA, sequestered within macrophages and neutrophils, was more successful at colonizing organs such as the kidney and brain (as well as being protected against vancomycin) than planktonic bacteria (33). The Agr-reverted phase variants identified in this study may play a role in this immune-mediated dissemination; Agr revertants would be able to survive phagocytosis and would hide within the Trojan horse as the phagocytic cells migrate away from the site of infection.

In summary, we show here that a fraction of Agr-negative strains are phase variants that arise by reversible genetic mutations in the *agr* locus either by alterations within short sequence repeats (SSR) or by site-specific rearrangements. To our knowledge, this is the first report demonstrating that Agr-negative strains can revert their Agr phenotype. Further, we discuss the possibility that the revertant cells serve as a cryptic strategy against the dead-end nature of infections favored by Agr-negative strains. These findings take us a step forward in understanding the infectious lifestyle of S. aureus and provide a new outlook on the prevalence of Agr-negative strains in the clinical setting. More extensive studies with clinically relevant samples, along with in-depth genotyping of any Agr-negative clinical samples identified as being reversible, are required to assess the prevalence and impact of Agr reversion in the health care setting.

## MATERIALS AND METHODS

### Bacterial strains and growth conditions.

Bacterial strains and plasmids used in this study are listed in Table 2. Strains were routinely grown in tryptic soy broth (TSB) at 37°C with shaking at 180 rpm. A total of 12.5 μg/ml of chloramphenicol (Cm12.5) was added when necessary.

**TABLE 2:** Staphylococcus aureus strains and plasmids used in this study

Strain or plasmid	Relevant characteristics	Reference or source
S. aureus strains		
RN4220	Derivative of 8325-4, restriction minus, modification plus	42
MW2	Community-acquired MRSA	43
MW2 Δ*agr*	Isogenic MW2 mutant lacking the whole *agr* locus	This study
RAV_MW2_	Reversible Agr variant derived from MW2	This study
AV1_MW2_	Irreversible Agr mutant derived from MW2	This study
AV2_MW2_	Irreversible Agr mutant derived from MW2	This study
AV3_MW2_	Irreversible Agr mutant derived from MW2	This study
AV4_MW2_	Irreversible Agr mutant derived from MW2	This study
s0437	Clinical isolate, MSSA	41
RAV_s0437_	Reversible Agr variant derived from s0437	This study
RAV(66r)	Clinical MRSA isolate, reversible Agr mutant	This study
MW2 P3*venus*	MW2 carrying the pRIT_RNAIII_*venus* Agr reporter plasmid	This study
MW2 ∆*agr* P3*venus*	MW2 Δ*agr* carrying the pRIT_RNAIII_*venus* Agr reporter plasmid	This study
RAV_MW2_ P3*venus*	RAV_MW2_ carrying the pRIT_RNAIII_venus Agr reporter plasmid	This study
Plasmids		
N315 pRIT*asp-gfp*	N315 carrying the pRIT_asp_*gfp* SigB reporter plasmid	38
N315 Δ*sigB* pRIT*asp-gfp*	N315 *sigB* deletion strain carrying the pRIT*asp-gfp* SigB reporter plasmid	This study
pRIT5H	Shuttle vector, used to make pRIT derivatives, Cm^r^	41
pRIT_RNAIII_*venus*	Agr P3 promoter-*gfp* transcriptional fusion	This study
pRIT*asp-gfp*	P*asp23*-*gfp* transcriptional fusion, SigB reporter	38

Strains MW2 and s0437 were used to generate *in vitro* Agr-negative mutants and to test their reversibility. Strain N315 was used in some fluorescence experiments.

A total of 173 clinical S. aureus samples, comprising 74 MRSA and 99 methicillin-susceptible S. aureus (MSSA) isolates, were collected from the Kanto region of Japan. Cells were picked from pure cultures on Mueller-Hinton agar plates and grown in TSB before making glycerol stocks.

### Construction of *agr* deletion mutant.

An *agr* deletion mutant was constructed by double-crossover homologous recombination using the pMAD-tet vector as described previously (38, 39). Briefly, the upstream and downstream regions flanking the entire *agr* locus were amplified using primer pairs agr-up-f(BamHI) and agr-up-r(BamHI) and agr-down-f(ecoR1) and agr-down-r(bgl2) (Table 3) and then cloned into pMAD-tet to generate pMAD-tet-Δ*agr*. This plasmid was introduced into strain MW2 by phage transduction after passaging through strain RN4220, and the *agr* mutant was constructed as previously described (39). The absence of the *agr* locus was confirmed by lack of hemolysis.

**TABLE 3:** Primers used in this study

Primer	Sequence (5′–3′)
agr front(RNA3)	AGTTGGGATGGCTTAATAAC
agr back	CAGCTATACAGTGCATTTGC
agr4	CCGGTCTTCGAGACTATTTC
agr5	AAGCCTATGGAAATTGCCCT
agr-up-f(BamHI)	TATGAGGATCCAAATTTATCAATTACCGA
agr-up-r(BamHI)	TTAAGGGATCCCAACTTAATAACCATGTA
agr-down-f(ecoR1)	GGCGAATTCAATTGTAAATCTTGTTGG
agr-down-r(bgl2)	TCAGATCTTTACGAAGCAAATTGGTGGC

### Variant generation.

Agr-negative variants were generated from two S. aureus strains: MW2 and s0437. Independent cultures of MW2 and s0437 were grown overnight in TSB to stationary phase and then subcultured at a 1,000-fold dilution into fresh TSB, TSB with 5% human blood, or TSB with 5% serum. Blood was mixed in a 3:2 ratio with filter-sterilized anticoagulation solution. The anticoagulation solution consisted of 0.32% (wt/vol) citric acid monohydrate, 0.88% (wt/vol) trisodium citrate dihydrate, and 0.88% (wt/vol) glucose prepared in Millipore water. Subcultures were repeated up to 3 to ∼7 times, and samples were plated onto sheep blood agar (SBA) at each stage to screen for nonhemolytic colonies.

### Revertant generation.

Agr-negative variant strains derived from MW2 and s0437 were grown overnight in TSB in 3 independent cultures to stationary phase. Samples were subcultured at a 1,000-fold dilution into fresh TSB and grown to stationary phase. Subcultures were repeated up to 4 times, and serial dilutions of the samples were plated onto SBA at each point to screen for hemolytic colonies.

A total of 61 of the 173 clinical isolates were characterized as being Agr negative by the CAMP test. All 61 Agr-negative strains were tested in an initial revertant generation screening. A single culture of each strain was grown overnight in TSB to stationary phase. Samples were subcultured at a 1,000-fold dilution into fresh TSB and grown to stationary phase. Subcultures were repeated up to 4 times, and serial dilutions of the samples were plated onto SBA at each point to screen for hemolytic colonies.

### Assessment of Agr activity on sheep blood agar plates.

Traber et al. demonstrated that a variation of the CAMP test can be used to assess Agr activity of S. aureus by streaking test samples perpendicularly to an S. aureus strain that produces only β hemolysin and analyzing the resulting patterns of hemolysis for the distinct pattern of Agr-controlled δ hemolysin (8, 40). Thus, the CAMP test was used to assess Agr activities of the isolated variant and revertant strains. An overnight culture of strain RN4220 (produces only β hemolysin) grown in TSB was streaked down the center of an SBA plate using a cotton swab, and the plate was incubated at 37°C for 4 to 6 h. Following this, test samples and controls (strain MW2 and its isogenic Δ*agr* mutant) were streaked perpendicularly to RN4200, approaching the streak but not coming into direct contact with it. Plates were incubated at 37°C for 12 to 16 h, until the hemolysis patterns of the controls became clearly visible. Further incubation at 4°C was carried out, if necessary, to enhance hemolysis.

### *agr* locus sequencing.

Genomic DNA was purified using standard procedures. The whole *agr* locus encompassing *hld* (NCBI gene identifier [ID] 1004072, Staphylococcus aureus subsp. *aureus* MW2) through *agrA* (NCBI gene ID 1004076, Staphylococcus aureus subsp. *aureus* MW2) was amplified by PCR using the primers agr front and agr back (Table 3) and subjected to direct sequencing using the agr4, agr5, and agr back primers (Table 3) (Fasmac, Japan). The sequence data were analyzed using the DNASTAR sequence analysis suite.

### Plasmid construction.

The pRITP3_(RNAIII)_-*venus* Agr reporter plasmid was constructed by Life Technologies, Japan. This plasmid has the P3 promoter region of RNAIII (−181 to +12 from the transcription initiation site), the *venus* fluorescent protein coding region, and the terminator region from *clpB* cloned into the pRIT5H backbone (41).

### Measurement of reversion frequency.

Glycerol stocks of MW2, RVA_MW2_, MW2 Δ*agr*, and RAV(66r) were inoculated in fresh TSB in 10 separate test tubes. Test tube cultures were grown to stationary phase and then subcultured at a 1,000-fold dilution into fresh TSB. Subcultures were successively repeated 4 times in total. Serial dilutions from the final stationary-phase subculture were plated on SBA, and plates were incubated for 24 h at 37°C, after which the reversion frequency was scored as the percentage of hemolytic colonies on the final SBA plates.

### Monitoring the population-wide Agr activity.

S. aureus strains MW2 P3*venus*, MW2 Δ*agr* P3*venus*, AV1_MW2_ P3*venus*, RAV_MW2_ P3*venus*, N315 pRIT*asp-gfp*, and N315 Δ*sigB* pRIT*asp-gfp* were grown overnight in TSB medium with Cm12.5. The *asp-gfp* reporter was used to detect σ^B^ activity. The number of cells was adjusted to 1 × 10^9^ CFU/ml by referencing the optical density at 600 nm (OD_600_). Each WT was mixed with its respective mutant to obtain a series of samples with final WT proportions being 0%, 0.1%, 1%, 10%, and 100%. The samples were incubated at 37°C with shaking at 180 rpm. At 0, 2, 4, and 8 h, cells were observed with a fluorescence microscope (FSX100; Olympus, Center Valley, PA) and the frequency of fluorescent cells was determined. To confirm that Agr-positive WT cells were not lost during the experiment, samples from the 8-h time point were plated on SBA and the frequency of hemolytic colonies was determined. Additionally, 5-μl volumes of the mixed samples were spotted onto TSA with Cm12.5 and were incubated overnight at 37°C. AV1_MW2_ P3*venus* and RAV_MW2_ P3*venus* samples were also spotted onto TSA with Cm12.5 in a similar manner. Following incubation, colonies were viewed under a stereomicroscope to check for fluorescence.

### Phagocytosis assay.

The mouse macrophage line RAW 264.7 (American Type Culture Collection) was maintained in suspension culture in RPMI 1640 medium supplemented with penicillin (100 U/ml), streptomycin (100 U/ml), l-glutamine (2 mM), and 10% (vol/vol) heat-inactivated fetal bovine serum (FBS; Equitech-Bio) at 37°C with 5% CO_2_.

RAW 264.7 cells were seeded in a 16-well plate (0.5 × 10^4^ cells/well, 200 μl) and were grown overnight until reaching a density of 1 × 10^4^ per well. The cell culture medium was replaced with drug-free RPMI 1640 medium supplemented with glutamine and 5% FBS 1 h before the infection.

MW2 P3*venus*, RAV_MW2_ P3*venus*, and MW2 Δ*agr* P3*venus* were grown overnight in TSB with Cm12.5. Cells were harvested by centrifugation (6,000 rpm, 2 min, and 4°C) from overnight culture and resuspended in RPMI 1640 medium. Cell density was adjusted to 1 × 10^9^ CFU/ml by referencing the OD_600_ value. RAW 264.7 cells were infected with 10 μl of the bacterial cells at a multiplicity of infection (MOI) of ∼10. At 1 h postinfection, the culture medium was replaced with RPMI 1640 medium supplemented with glutamine, 5% FBS, and gentamicin (50 μg/ml) and the samples were incubated for 1 h to kill nonphagocytosed bacteria. Following this, the medium was once again replaced with drug-free RPMI 1640 medium supplemented with glutamine and 5% FBS. Samples were prepared for microscopy at 1, 5, and 9 h postinfection by removing the supernatant and fixing the cells with 200 μl of methanol. Images were captured using a light microscope (FSX100; Olympus, Center Valley, PA) and viewed with FSX-BSW software (Olympus). To check whether the presence of RAW 264.7 cells affected Agr reversion frequency, bacteria were collected from a separate set of samples after the final time point by lysing macrophages with 200 μl of cold PBS supplemented with 0.02% Triton X-100 and mixing with a pipette. These samples were then plated on SBA and the frequency of reversion was assessed by the frequency of hemolytic colonies and compared to that of the preinfection samples.
