# Supplementary figures and images for: Finding of Agr Phase Variants in Staphylococcus aureus
Source: mBio. 2019 Aug 6;10(4):e00796-19. doi: 10.1128/mBio.00796-19 (PMC6686034; doi:10.1128/mBio.00796-19)

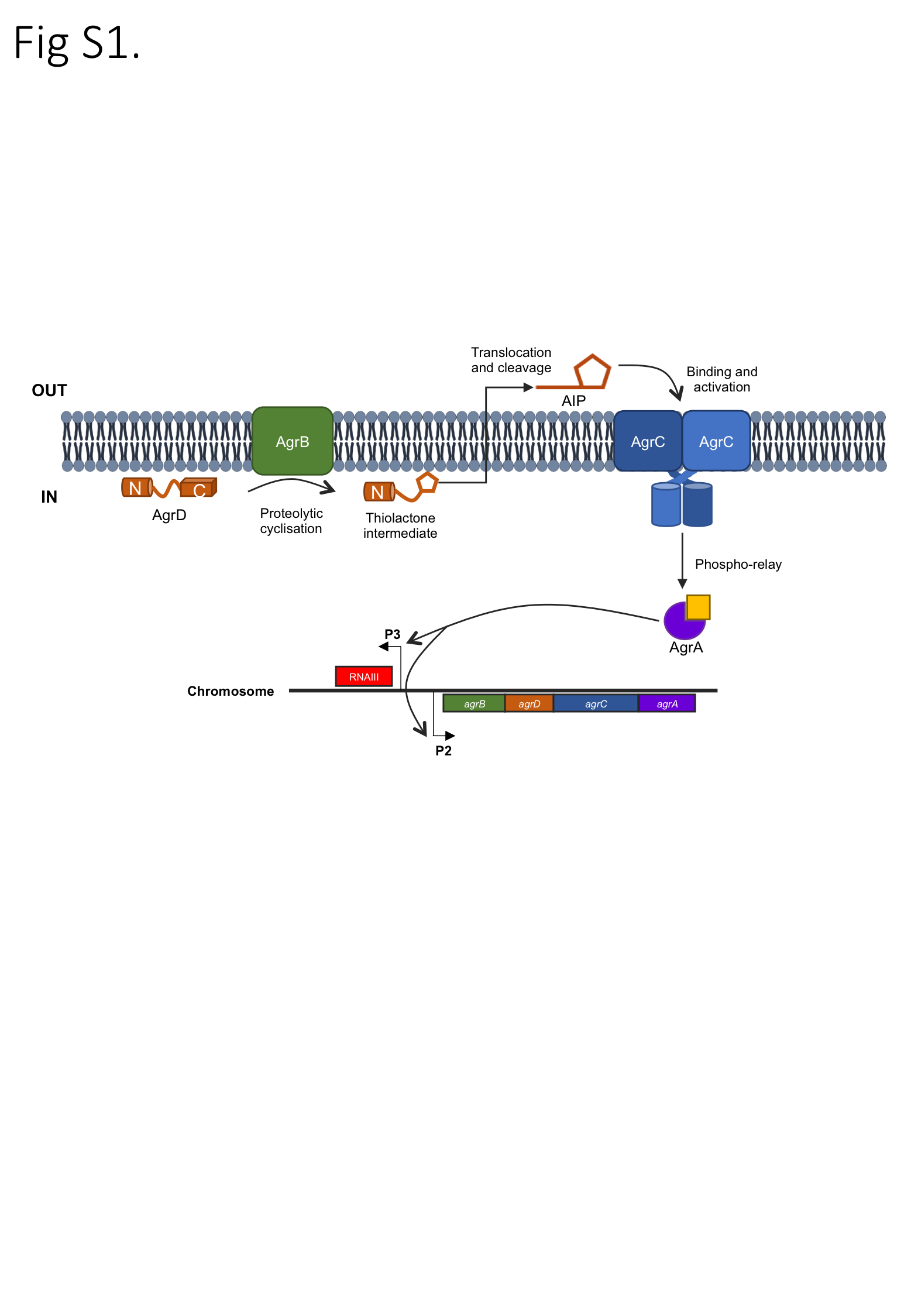

Supplement: FIG S1 [file mBio.00796-19-sf001.tif]
